# Widespread Progressive Telangiectasia in a 54-Year-Old Caucasian Male

**DOI:** 10.7759/cureus.94588

**Published:** 2025-10-14

**Authors:** Stefani Kikina, Layla Ali, Samuel Audet, Elena Koleva, Ayesha Imran, Georgia Morley, Hayder N Alhameedi

**Affiliations:** 1 Cardiology, Antrim Area Hospital, Antrim, GBR; 2 Dermatology, University College London Hospital, London, GBR; 3 Dermatology, Royal Berkshire Hospital, Reading, GBR; 4 Dermatology, Mid and South Essex NHS Foundation Trust, Basildon, GBR; 5 Dermatology, Queen Elizabeth Hospital, London, GBR; 6 Internal Medicine, Southampton General Hospital NHS Foundation Trust, Southampton, GBR

**Keywords:** alcohol, cutaneous vascular, photodermatology, telangiectasia, telangiectasia macularis multiplex acquisita, tmma, uv

## Abstract

Telangiectasia macularis multiplex acquisita (TMMA) is a rarely reported cutaneous vascular disorder characterised by widespread telangiectatic macules, most commonly affecting middle-aged adults. It has been reported predominantly in East Asian populations, particularly in Chinese and Korean cohorts. TMMA has also been observed in association with systemic conditions, including chronic liver disease, hypertension, type 2 diabetes mellitus, and dyslipidaemia. We present a case of a 54-year-old Caucasian male with progressive, symmetrical telangiectatic lesions affecting the face, upper limbs, and trunk. He reported chronic elevated alcohol intake and a history of significant sun exposure. Laboratory investigations were unremarkable. Histopathological examination confirmed superficial dermal telangiectasia without mast cell proliferation, consistent with TMMA. The patient was referred to a photodermatology specialist and managed conservatively with lifestyle modification. This case contributes to the limited English-language literature on TMMA and underscores the importance of distinguishing it from mimics such as telangiectasia macularis eruptiva perstans (TMEP).

## Introduction

Telangiectasia macularis multiplex acquisita (TMMA) is a rarely reported, acquired cutaneous vascular disorder characterised by widespread telangiectatic macules and patches. It most often affects middle-aged adults and has been reported predominantly in Chinese and Korean populations, with a notable male predilection [1-3]. Although TMMA is typically benign and asymptomatic, it has been associated with chronic liver disease, hypertension, type 2 diabetes mellitus, and dyslipidaemia [1,3,4].

The pathogenesis of TMMA is not fully understood. Proposed contributing factors include vascular fragility associated with ageing, alcohol-induced vasodilatation, and ultraviolet (UV) light-mediated dermal damage, including degeneration of elastic fibres and connective tissue [1,3,5]. Accurate diagnosis is essential, as TMMA must be distinguished from other causes of generalised telangiectasia, particularly telangiectasia macularis eruptiva perstans (TMEP), a rare form of cutaneous mastocytosis that may resemble TMMA clinically but differs histopathologically and in its systemic implications [6,7].

We present a case of biopsy-confirmed TMMA in a middle-aged Caucasian man, highlighting the clinical and histological features that supported the diagnosis and the importance of distinguishing it from mimics such as TMEP.

## Case presentation

A 54-year-old Caucasian male was referred to our dermatology clinic with a longstanding history of progressively worsening telangiectasia affecting multiple body regions over a 15-year period. He was otherwise healthy, with no significant past medical history and no family history of similar skin findings or systemic telangiectatic disorders. He did not smoke and reported consuming approximately 30 units of alcohol per week over many years. Although his occupation was office-based, he described substantial recreational UV exposure in earlier decades, including frequent sunbed use in his 20s and multiple annual sun holidays without consistent photoprotection.

**Figure 1:**
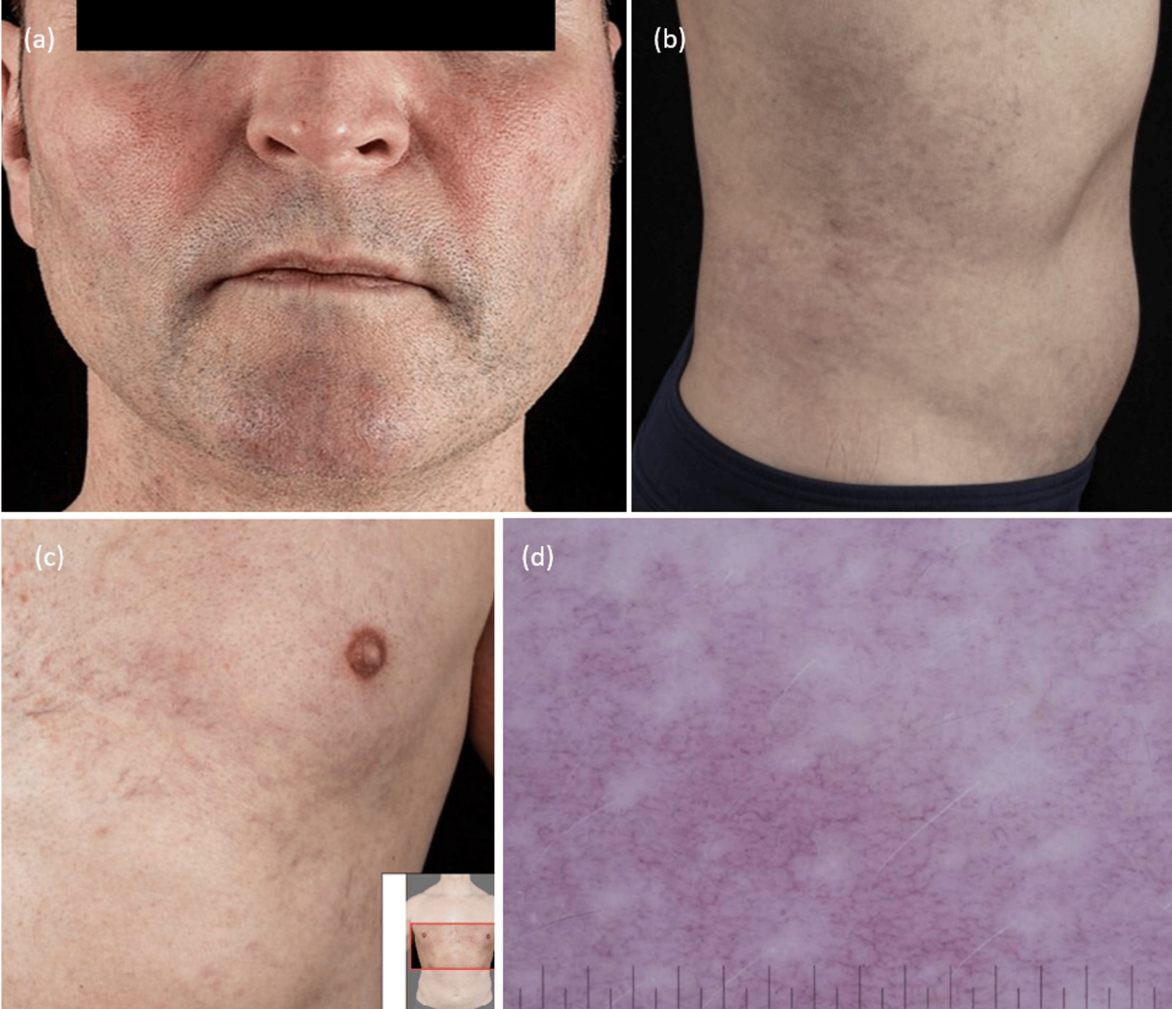
(a) Pronounced telangiectasia distributed bilaterally over the infraorbital and zygomatic regions, with associated erythema extending across the chin. (b) Telangiectatic macules were observed bilaterally on the lower flanks, more prominent on the right side. (c) Telangiectasia extending across the anterior chest wall with a well-defined erythematous base. (d) Dermoscopic image of the right flank revealing a dense network of dilated capillaries arranged in a reticular pattern, with a prominent erythematous background.

On examination, numerous telangiectatic macules and patches with an erythematous base were observed. The lesions were most prominent on the face, symmetrically distributed over the malar and infraorbital regions, with sparing of the nasal bridge (Figure 1). Additional telangiectases were noted on the lateral upper arms and shoulders, the dorsal forearms, more marked on the left, the upper chest in a V-shaped pattern, and scattered over the flanks and abdomen. No palpable nodules or plaques were present, and there was no urtication on rubbing the lesions. The remainder of the cutaneous examination was unremarkable, with no mucosal involvement or other vascular skin lesions such as spider angiomas or haemangiomas. Systemic clinical examination revealed no features suggestive of ataxia, sclerodermatous change, or bleeding tendency.

Comprehensive clinical assessment and routine blood tests were unremarkable. Screening for hepatitis B and C, human immunodeficiency virus, and connective tissue diseases such as antinuclear antibody, extractable nuclear antigen panel, rheumatoid factor, and antineutrophil cytoplasmic antibody was negative. The patient was referred to a photodermatology specialist for further evaluation. Two 4-mm punch biopsies were taken from telangiectatic lesions on the right flank to confirm the diagnosis.

Histopathological findings

**Figure 2:**
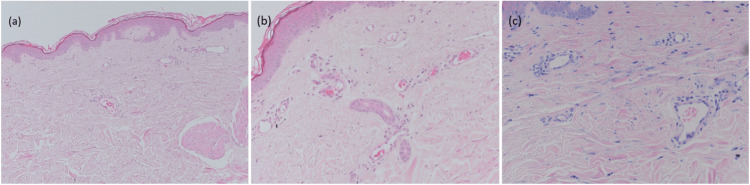
(a) Four-mm punch biopsies from the right flank were examined under 10x magnification, (b) 20x magnification, and (c) 40x magnification.

Skin punch biopsies from the right flank were taken, and histopathological examination revealed telangiectasia with minimal perivascular chronic inflammation (Figure 2). Direct immunofluorescence was unremarkable, showing no immune complex deposition. Mast cell staining with toluidine blue and anti-tryptase immunohistochemistry revealed no increase in mast cell density or clustering around the capillaries, which helped exclude TMEP. The findings were consistent with a diagnosis of TMMA.

Haematoxylin and eosin staining revealed a normal epidermal architecture with telangiectasia in the dermis, characterised by dilated capillaries and venules. There was minimal perivascular chronic inflammation, consisting of scanty inflammatory cells. The overall inflammatory pattern remained nonspecific, with no significant epidermal infiltration or structural alteration observed.

## Discussion

TMMA is a rarely reported cutaneous vascular disorder characterised by widespread, persistent telangiectatic macules in the absence of systemic involvement. It was first defined as a distinct clinical entity in 2012, with diagnostic criteria including the following: clusters of telangiectasia superimposed on erythematous macules, affecting the upper limbs, chest, back, or thighs; absence of mucosal or systemic involvement; absence of autoimmune disease; absence of ataxia [1].

The condition has been reported almost exclusively in Chinese and Korean males between the third and sixth decades of life [1-3]. It has been observed at higher rates in patients with systemic conditions, such as viral hepatitis, type 2 diabetes mellitus, dyslipidaemia, and hypertension, suggesting possible underlying metabolic and vascular contributions to its development [1,3,4]. The underlying pathogenesis of TMMA remains poorly defined. Proposed contributors include chronic ultraviolet-mediated dermal injury, alcohol-induced vasodilatation, and possible structural alterations of dermal vasculature, although these mechanisms have not been conclusively established [1,3,5].

Although TMMA is typically asymptomatic, its clinical appearance may prompt concern, particularly due to its widespread and persistent nature. Crucially, it must be differentiated from other causes of generalised telangiectasia, including hereditary haemorrhagic telangiectasia, ataxia-telangiectasia, systemic sclerosis, and generalised essential telangiectasia. In each of these, mucosal, systemic, or progressive internal involvement is common, and clinical evaluation must seek to exclude such features [1,3]. In our case, there were no systemic symptoms, and thorough laboratory, radiological, and serological investigations excluded autoimmune, infective, or malignant associations.

Another important differential diagnosis remains TMEP, a rare form of adult-onset cutaneous mastocytosis. Although TMEP may present with similar telangiectatic macules, it is histologically characterised by increased mast cell infiltration in the papillary dermis. Mast cell-specific stains, including toluidine blue and anti-tryptase immunohistochemistry, typically reveal increased mast cell density and perivascular clustering [6]. TMEP may also exhibit a positive Darier’s sign and can be associated with systemic mastocytosis or haematological malignancies, with important diagnostic and prognostic implications [6,7]. In our case, mast cell staining demonstrated no increase or clustering, effectively excluding TMEP.

Several reports have described TMMA in association with chronic liver disease, particularly in patients with viral hepatitis B or C [1,4,5]. Cutaneous vascular findings in liver disease, such as spider naevi and palmar erythema, have been attributed to hyperestrogenism, altered hepatic metabolism, and increased vascular endothelial growth factor (VEGF) expression. TMMA has been reported in similar clinical contexts and considered a potential cutaneous manifestation within this spectrum of hepatic vasculopathy [4,5]. In the present case, liver function tests and viral serologies were within normal limits, and abdominal ultrasonography revealed no hepatic abnormalities. This suggests that TMMA may arise in the absence of overt liver disease. Notably, the patient reported longstanding alcohol consumption and substantial cumulative light exposure, both of which may contribute to dermal vascular changes. Alcohol has recognised vasodilatory effects, while chronic UV radiation can result in dermal elastosis, vascular ectasia, and connective tissue degeneration [5].

There are currently no established guidelines for the treatment of TMMA. Given its benign course, management is generally conservative and individualised. Cosmetic camouflage may suffice for patients with mild symptoms, while pulsed dye laser and other vascular-targeted therapies may be considered for those seeking aesthetic improvement. In our case, the patient declined laser therapy but undertook behavioural modifications, including daily use of broad-spectrum SPF50 sunscreen and reduction of alcohol intake to within national recommended limits. At 12- and 24-month follow-up, no further progression or new lesions were noted.

This case illustrates the importance of a comprehensive approach in patients presenting with generalised telangiectasia. Excluding systemic, autoimmune, or haematological causes is essential, particularly in rare disorders such as TMMA that share clinical overlap with more serious conditions. It also highlights the growing recognition of TMMA as a distinct diagnostic entity, with potential relevance beyond East Asian populations. As additional cases emerge, further insight will be gained into its pathogenesis, systemic associations, and optimal management.

## Conclusions

TMMA remains a rarely reported but distinct cutaneous vascular entity, with limited representation in English-language literature. This case contributes to the evolving clinical understanding of TMMA by highlighting its characteristic features, the importance of thorough exclusion of systemic and haematological causes of telangiectasia, and its differentiation from mimics such as TMEP. Although asymptomatic, TMMA may cause cosmetic concern and warrants a holistic, patient-centred approach. In this case, lifestyle modification, including reduced alcohol intake and consistent photoprotection, was associated with disease stability over two years. Continued reporting of TMMA cases will be essential to improve diagnostic confidence, explore potential associations, and refine future treatment pathways for this uncommon condition.
